# HSA: A Heuristic Splice Alignment Tool

**DOI:** 10.1186/1752-0509-7-S2-S10

**Published:** 2013-12-17

**Authors:** Jingde Bu, Xuebin Chi, Zhong Jin

**Affiliations:** 1 Supercomputing Center, Computer Network Information Center, Chinese Academy of Sciences, Beijing, China; 2 Graduate University of Chinese Academy of Sciences, Beijing, China

## Abstract

**Background:**

RNA-Seq methodology is a revolutionary transcriptomics sequencing technology, which is the representative of Next generation Sequencing (NGS). With the high throughput sequencing of RNA-Seq, we can acquire much more information like differential expression and novel splice variants from deep sequence analysis and data mining. But the short read length brings a great challenge to alignment, especially when the reads span two or more exons.

**Methods:**

A two steps heuristic splice alignment tool is generated in this investigation. First, map raw reads to reference with unspliced aligner - BWA; second, split initial unmapped reads into three equal short reads (seeds), align each seed to the reference, filter hits, search possible split position of read and extend hits to a complete match.

**Results:**

Compare with other splice alignment tools like SOAPsplice and Tophat2, HSA has a better performance in call rate and efficiency, but its results do not as accurate as the other software to some extent.

**Conclusions:**

HSA is an effective spliced aligner of RNA-Seq reads mapping, which is available at https://github.com/vlcc/HSA.

## Background

With next generation sequencing technology has made steady progress [1-3], both time and money costs on sequencing have decreased dramatically in recently. It means that we can obtain big sequencing data in a very short time. It is really good news to biological research, almost every branch of biology could benefit from the ex-scale data mining of genomics, transcriptomics, epigenetics, and so on. In the other side of this coin, big sequencing data processing is becoming a new bottleneck and costs great amount of manpower [3-8]. RNA-Seq [2] is a next generation transcriptomics sequencing technology sequencing whole transcriptome data. Due to the big data property, much comprehensive RNA information like expression of genes or splice variants are available for us to investigate. Compare with previous RNA research method, expressed sequence tags (ESTs) or microarray, the advantage of RNA-Seq method is lower sequencing cost and big data. More and more researchers select RNA-Seq technology as their main method in transcriptomics research [9]. However, the bioinformatics-analyzing barrier for NGS data also exists in RNA-Seq data processing.

Splice junctions are points on a DNA strand at which an intron is cut out in gene expression, right before the transcripts are translated into protein [10]. It means that RNA-Seq reads may span two or more exons, reads coming from splice junction region is steady increasing with RNA-Seq read length expanding. This situation exacerbates the difficulty of short reads data mining. Short reads mapping always carry a crucial role in sequencing data processing pipeline, especially when we process RNA-Seq data with de novo assembly method. But most short read assemblers do not track each individual read back to the assembly [11]. It is a big challenge in designing an efficiently program to finish reads mapping and splice junctions finding with high throughput sequencing data as well as combining with genome region location.

Based on whether the aligner support splice reads alignment, we can divide aligner into two categories: unspliced aligner and spliced aligner. Unspliced aligner can handle common reads which come from major part of genome, certainly allow reads have errors or indels, the highest-profile software are BWA [11], Bowtie [12], SOAP2[13], and so on. Spliced aligner can support common and splice junction reads, which can provide specified results in genome structural annotation [14]. Several professional spliced aligners designed their algorithms by consulting different strategies, like statistical way or divide and conquer principle etc. Tophat[15] is the most common tool for spliced mapping by means of using an 'Exon-fist' method [7], this method contains two steps: first, mapping read with Bowtie and assembling the mapped reads into consensus by MAQ [16]; second, splice junctions are generated from adjacent exons, and the unmapped reads (IUM reads) are mapped to the joint sites. Hence, Tophat is suitable for detecting splice junctions with high sequencing coverage. Q-PALMA[17] is an implement of statistical way, it detects splice junctions using machine-learning method and predict splice sites using mapped reads as training model. Meanwhile, biases have been brought in the predicted splice sites when model training carried out. Divide and conquer is also a major strategy for spliced aligner algorithm designer to choose. All of SpliceMap [18], MapSplice [19] and SOAPsplice [20] are chosen it as their start point of designing a spliced alignment pipeline, but their implement of the strategy is totally different. SpliceMap divides IUM reads into two halves, and filter unspliced aligner mapping results of each half by paired-ends information and canonical form of intron. Unlike SpliceMap, MapSplice splits IUM reads into several segments, and searches spliced sites from mapped segments, finally merges all segments results. SOAPsplice divides IUM reads shorter than 50 bp into two segments, which is similar to SpliceMap. The other IUM reads are split into multiple segments, and known splicing motifs are used to filter mapping result. Different divide methods have different effect on accuracy, call rate and difficulty of subsequent processing: too little segments may reduce call rate; too many segments may decrease accuracy; too short segment may have multiple aligned result, which is hard to identify. In addition, gapped alignment is more important than ungapped alignment in variant discovery [11].

In brief, we find that the crucial points are how to design an appropriate IUM reads division strategy and how to search the spliced site from the segments mapping results. Here, we introduce a heuristic method based spliced aligner special for detecting splice junctions, named HSA. A two-step approach is carried out to search splice junctions, which contain both nonconservative and conservative motifs. The first step, we use BWA as an unspliced aligner to map reads; the second step is heuristic alignment stage, where a seed and extension strategy is applied for spliced alignment. We pick trichotomy as division strategy; three equal segments are regard as seeds and the unspliced mapping are performed. We try to search splice site and finish spliced mapping by filtered segments results. Gapped alignment is allowed in the two stages. Performance measurement comparison is carried out among HSA, SOAPsplice, Tophat2, MapSplice and SpliceMap, based on the simulated data sets. We evaluate from three angles, cost (running time), sensitivity (call rate) and accuracy. HSA shows better performance in the first two respects with an acceptable accuracy.

## Methods

### Heuristic algorithm

Heuristic technique is designed for solving a problem quicker when classic methods are too slow, by trading optimality, completeness, accuracy, and/or precision for speed [21]. Seed and extend strategy is one of the most popular heuristic algorithms; here we use it to solve spliced alignment problem. IUM reads are split into three equal segments working as seeds, and then they are aligned to the reference; candidate seeds alignment results are examined with more sensitive criteria; iterative extension and merging of initial seeds determine the exact spliced alignment for the read.

### Pipeline

HSA takes two stages of process to map RNA-Seq reads to the reference, so called normal alignment stage and heuristic alignment stage. The details are shown as follow.

#### (1) Normal alignment stage

HSA aligns the original reads by means of BWA, a popular unspliced aligner. Bi-direction BWT [22] is used to index the reference sequence, and each sequence is scanned from both 5' and 3' ends. Breadth first search (BFS) is carried out to consider mismatch and gap; user could configure the tolerance number of mismatch and gap. IUM reads generated from this process will be put into the next stage, heuristic alignment stage.

#### (2) Heuristic Alignment stage

**Figure 1:**
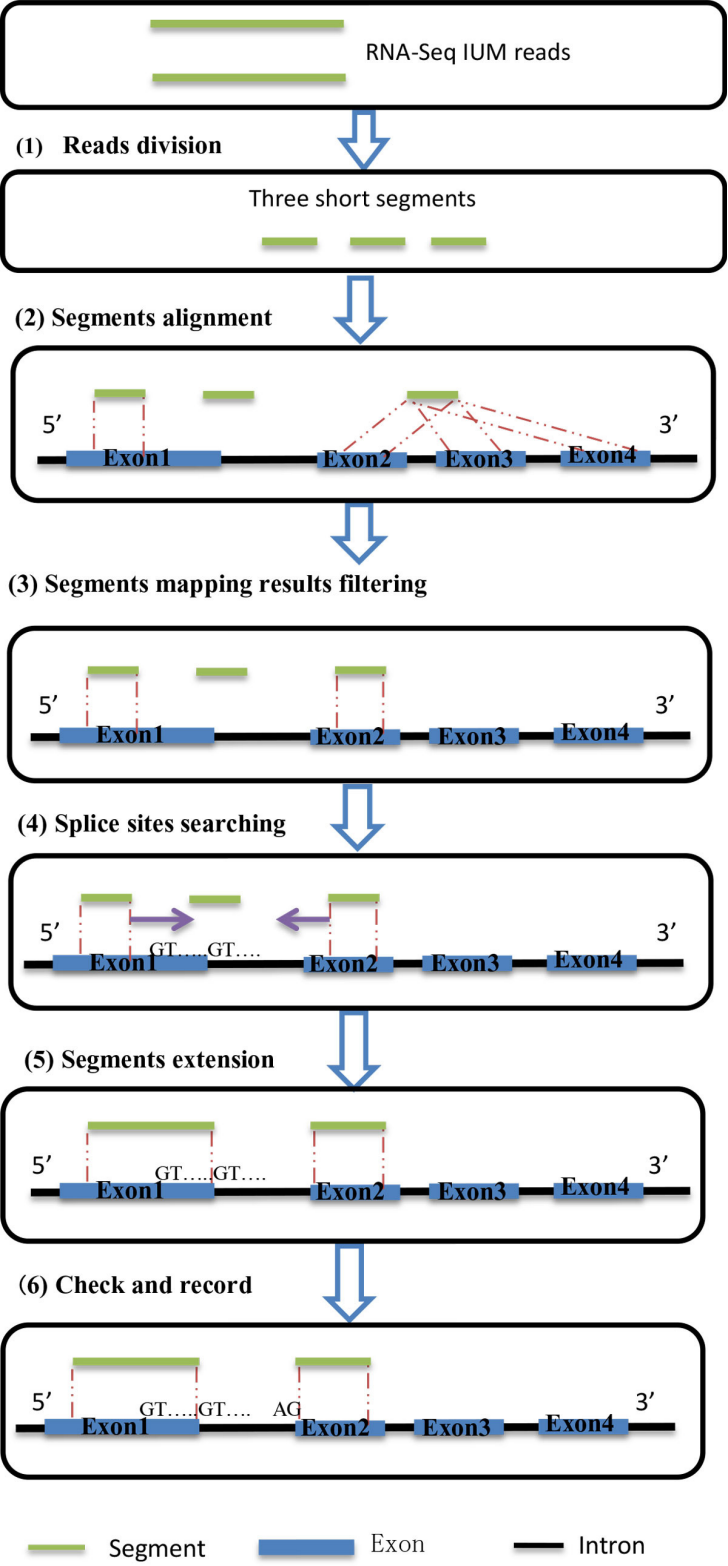
**Pipeline of splice alignment**. Splice alignment contains six steps. We use trichotomy as divide method, three equal segments are regarded as seeds and finish unspliced mapping by BWA - an unspliced aligner. Segments results are filtered based on the mapping results and known splice junctions' information. Then, we try to search splice sites based on mapped segments information and known splicing motifs. Splice alignment will be finished after segments extension, and all splice sites are recorded. Gapped alignment is allowed in both initial alignment stage and heuristic alignment stage.

A seed and extension strategy is applied for heuristic alignment. The pipeline of this step is shown as Figure 1, containing six steps. Each IUM read are cut into three equal segments, which is considered as seeds in heuristic strategy. Here, all the segments are mapped to both positive and negative strands of the reference. We filter the mapped segments, which we call them hits, with the known splice junction information. After searching split positions of reads, we extend segments to a hypothetical junction site. Gapped alignment is allowed in both normal alignment stage and heuristic alignment stage.

Step 1: Reads division. All IUM reads longer than 60 bp are split into three equal segments, because too short and too many segments lead to inaccuracy of our filter result. These segments represent seeds in our algorithm.

Step 2: Segments alignment. BWA is used here to map all the segments with the permission of mismatches, but without the permission of gaps. And the number of mismatch is decided by RNA-Seq read error rate and segment length. At least two mapped seeds of three seeds are required in splice alignment (if all of them can mapped to reference, we will filter false seed alignment in next step).

Step 3: Segments mapping results filtering. Segments mapping results are multiple or false strand in some cases. Two-filter strategy is performed here. First, check all the overlapped mapped segments are on the same chromosome and strand; second, distance of two adjacent segments should be 50-50,000 bp, which may locate on two exons [18].

After all alignments are filtered, we will try another directional strand of the reference. Experiment results shows that the filter results have significant impact on leaving steps, because if seeds' alignment on the false strand of reference we may get a false splice junction.

Step 4: Splice sites searching. If at least two segments coming from the same IUM read are survived from the filtering, we select one of them as a seed to find possible splice sites. Canonical motif (GT-AG) and non-canonical motifs (GC-AG and AT-AC) are used for searching the candidate splice sites. We use the edit distance of read and reference sequence to filter candidate splice sites; this method helps us in finding the most accuracy splice sites.

Step 5: Segments extension. Seeds are extended to those candidate splice sites, and then checked if the motif type is match to confirm the splice sites. If the unmapped part is short, less than 10 bp, or the unmapped segment is the first or the last one, we use the longest extension as the splice alignment. If we do not have a suitable accuracy extension result, we will treat the read as nonconservative, and extend seeds freely, mismatch number is determinate by RNA-Seq read error rate and length, we will refine the freely extension result in result output stage. Mismatch and gap are allowed in the extension with default number of 3, and users can configure it by themselves.

Step 6: Check and record. All the splice sites are double checked with this data set to make sure and record.

### Implementation

HSA is written in C language and runs on Linux system. We choose bi-direction-BWT [22] as the index method and BWA as an unspliced aligner owing to its good performance on both mapping efficient and gap support. Now, HSA is open access at https://github.com/vlcc/HSA.

## Results

**Figure 2:**
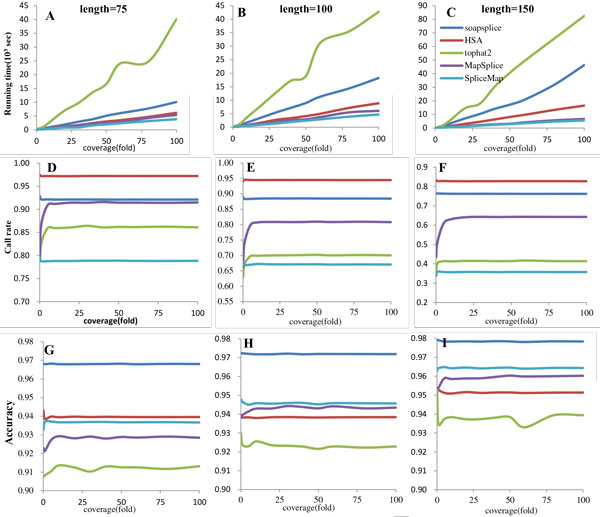
**Simulated dataset test result**. The statistical results on 75, 100 and 150 bp simulated reads in BAsplice, SOAPsplice, Tophat2, MapSplice and SpliceMap under different coverage of transcript. A, B and C show the running time results. D, E and F show the call rate results. G, H and I show the accuracy result. Three columns are the statistical results of 75, 100 and 150 bp simulated reads.

We extract 5153 transcripts and 17661 known splice junctions from human chromosome 17 downloaded from Ensemble database. All the transcripts are longer than 350 bp. We use wgsim [23] as read simulator to do a simulation test for HSA and four other spliced aligners, with mutation rate and base error rate and mutation rate of 0.001 and 0.02. Three read lengths, 75, 100 and 150 bp are used to measure the algorithm performance under different read length. To test the performance of algorithm on different coverage, we generate datasets with eleven kinds of coverage (0.1, 1, 5, 10, 20, 30, 40, 50, 60, 80 and 100 fold). The quality of methodology are measured in three ways, cost (shown as running time), sensitivity (shown as call rate) and accuracy. All statistical results are shown in Figure 2.

Figure 2A, B and 2C describe the running time cost by five software with three read lengths, 75, 100 and 150 bp, respectively. All these works have been done on the same platform (a single core of 2.8 G AMD Opteron 2220 processor, Centos 5.1 operation system). Tophat2 is always the top cost in all three figures. SOAPsplice follows Tophat2 and running time goes higher with increasing read length. HSA costs least at read length 75 bp together with MapSplice and SpliceMap, but goes up higher and much higher than those two aligners when comes to read length 100 and 150 bp.

Figure 2D, E and 2F shows the call rate results with three different read lengths. HSA performs best among all test aligners, follows by SOAPsplice, MapSplice, Tophat2 and SpliceMap. This trend always keeps in all three kinds of read lengths.

Although the call rate results decrease with read length increasing in all five software, HSA still holds great sensitivity with longer read length. For instance, the call rate value of HSA at the coverage of 30 fold are 97.21%, 94.47% and 82.77% for 75, 100 and 150 bp read length, while the call rate value of SOAPsplice are 92.11%, 88.48% and 76.26% and the call rate value of Tophat2 is 86.41%, 70.07% and 41.52%. One reason of HSA has the highest call rate is it supports nonconservative splice alignment.

Seen from Figure 2, all five aligners show great performance on accuracy, and all the test results are higher than 90%. With the read length increase, the accuracy value of all software increases, too. SOAPsplice get the top seat in all three read lengths. HSA follows SOAPsplice in 75 bp case, but falls behind SpliceMap and MapSplice in 100 and 150 bp cases.

In seed and extend strategy, seeds may have multi alignment that it's hard to exclude false result. Or the seed may be the splice junction region, and still have aligning result. This will reduce the accuracy of HSA, but it is fairly acceptable. And when we try to locate the major reason for accuracy reduce by checking false mapping results, we find that about 82% false results are caused by mapped to a reverse strand of the reference, which means the reference have duplicate regions on two complement strands. From the chart, we also find that accuracy of mapped reads increase when read length grows, but the call rate decrease. The increase of seeds uniqueness induces the decrease of seed alignment false rate. Another reason is human has much more short exons, 25% are shorter than 100 bp. That makes the read longer than 100 bp has a great probability to span more exons, which cause more difficulty to mapping. We abandon genes which exon length less than 100 bp, and test the tool again, the test result shows that the accuracy improved 4% (from 93.94% to 97.41%).

## Conclusions

HSA is an effective spliced alignment tool for RNA-Seq data, and it supports the alignment of both nonconservative and conservative alternative splicing. To make the mapping results available and compatible for other software, we pick SAM format [24] as standard to output results. Compare with other existing tool, HSA shows great performance on cost and sensitivity under different read lengths and sequencing depth. But HSA has a lower accuracy results based on the simulation test with the other five software. This is mainly because the existence of duplication region of gene, which leads to a false result when reads comes from the two complement strands. Compared with half divide strategy like MapSplice, short length of seed will reduce the iteration if we apply unspliced alignment allow mismatches and gaps. And if the read spans two exons, at least two of the seeds must have alignment result.

## Competing interests

The authors declare that they have no competing interests.

## Authors' contributions

Designed the experiments: ZJ. Draft the pipeline: JDB. Implement, simulation and test by. Wrote the paper: JDB, XBC and ZJ.

## Declarations

The publication of this article has been funded by the Chinese Academy of Sciences Cloud projects in Science and Technology (XXH12503-05-06) of China.

This article has been published as part of *BMC Systems Biology *Volume 7 Supplement 2, 2013: Selected articles from The 6^th ^International Conference of Computational Biology. The full contents of the supplement are available online at http://www.biomedcentral.com/bmcsystbiol/supplements/7/S2.
